# Shared Decision-Making in Acute Pain Services

**DOI:** 10.1007/s11916-023-01111-8

**Published:** 2023-05-08

**Authors:** Corina M. Bello, Simone Mackert, Michael A. Harnik, Mark G. Filipovic, Richard D. Urman, Markus M. Luedi

**Affiliations:** 1 Department of Anaesthesiology and Pain Medicine, Inselspital, Bern University Hospital, University of Bern, 3010 Freiburgstrasse Bern, Switzerland; 2 Department of Anaesthesiology Spital Grabs, Spitalregion Rheintal Werdenberg Sarganserland, Spitalstrasse 44, Grabs, St. Gallen 9472 Switzerland; 3 Department of Anaesthesiology, College of Medicine, The Ohio State University, Columbus, OH 43210 USA

**Keywords:** Acute pain, Shared decision-making, Patient-centred care, Patient satisfaction

## Abstract

**Purpose of Review:**

The implementation of shared decision-making (SDM) in acute pain services (APS) is still in its infancies especially when compared to other medical fields.

**Recent Findings:**

Emerging evidence fosters the value of SDM in various acute care settings. We provide an overview of general SDM practices and possible advantages of incorporating such concepts in APS, point out barriers to SDM in this setting, present common patient decisions aids developed for APS and discuss opportunities for further development.

**Summary:**

Especially in the APS setting, patient-centred care is a key component for optimal patient outcome. SDM could be included into everyday clinical practice by using structured approaches such as the “seek, help, assess, reach, evaluate” (SHARE) approach, the 3 “MAking Good decisions In Collaboration”(MAGIC) questions, the “Benefits, Risks, Alternatives and doing Nothing”(BRAN) tool or the “the multifocal approach to sharing in shared decision-making”(MAPPIN’SDM) as guidance for participatory decision-making. Such tools aid in the development of a patient–clinician relationship beyond discharge after immediate relief of acute pain has been accomplished. Research addressing patient decision aids and their impact on patient-reported outcomes regarding shared decision-making, organizational barriers and new developments such as remote shared decision-making is needed to advance participatory decision-making in acute pain services.

## Introduction

Medical decision-making has changed rapidly over the past few decades. Giving patients more control and choices in an attempt to support patient autonomy has led to a paradigm shift in medicine [1••]. Indeed, the clinician–patient relationship has evolved from a classic paternalistic model to an informative, interpretative and deliberative interaction between the costumer (patient) and the providers (healthcare professionals) [2]. Shared decision-making (SDM) even goes beyond such paradigms, changing the question of “what’s the matter with you” towards “what matters to you”, as Barry put it [3]. Shared decisions are thus based on “mutual respect and participation” [4]. Today, several international guidelines (e.g. from the National Institute for Health and Care Excellence (NICE) [5] or the Agency for Healthcare, Research and Quality (AHRQ) [6]) provide advice on how to incorporate such a model into everyday clinical practice [7]. Interestingly though, while the concept was rapidly incorporated into other areas of medicine, specific adaptations for the special setting of perioperative medicine or acute pain services (APS) has lagged behind [8, 9•]. Of note, in APS, which are mostly led by anaesthesia specialists, current guidelines do not provide advice on SDM, although they aim to assist “the practitioner and patient in making decisions about healthcare”, as stated in the first sentence of the guideline [10]. However, especially in the stressful setting acute pain relief with various available treatment options, making decisions based on a patient’s pain experience, values and expectations should represent the standard of care [11, 12••]. We provide an overview of current SDM practices in general; discuss prerequisites, advantages and limitations of its implementation specifically for APS; explore tools that help apply SDM in everyday practice of APS; and finally point out urgent gaps of knowledge and imminent challenges regarding SDM in the modern APS setting.

## The Evolution of SDM and Its Role in the Development of Acute Pain Services (APS)

SDM is not a new concept. A first conceptualization was proposed by Cathy Charles already in 1997: at least two people (doctor and patient) are involved in the decision-making process, both parties exchange information, both doctor and patient are determined to find a consensus regarding treatment, and agreement is reached on the treatment to be carried out [13]. More recently, SDM has been defined as “a collaborative process involving a person and their healthcare professional working together to reach a joint decision about care” [5]. Especially in settings where patients are offered (and regularly left with) numerous choices regarding evidence-based treatment options with equivalent predicted outcomes [14], SDM is gaining increased importance and is even considered a quality indicator of a healthcare system in terms of patient-centred care [14, 15].

The concept of APS, on the other hand, was developed in response to the lack of a perioperative pain management system [16, 17] and its resulting consequences, such as longer hospital stays or prolonged recovery time [18]. Since the 1980s, many European and US-based hospitals have started to offer APS in order to alleviate acute postoperative pain requiring specialist skills, such as peridural anaesthesia [19]. Currently, the role is shifting towards supporting the care of patients with pre-admission chronic pain, addictions (such as substance or more specifically opioid abuse), complex psychosocial or physical comorbidities (such as cancer) by providing multimodal pain management strategies to patients suffering from breakthrough cancer pain or pain after acute trauma [9•, 20]. Training, education of staff, organization of services or the development of standard protocols regarding the management of pain and finally quality control on an institutional level may also be attributed to APS [9•, 18]. While APS require interdisciplinary collaboration, the “anaesthesia-driven” model of APS remains the most common in Europe and the USA — the integration of professionals with different medical backgrounds (including physiotherapists, surgeons, nurses, oncologists, geriatric or paediatric specialists) in APS remains a challenge [19, 21].

The diversity of causes for acute pain and also the complexity of each patient’s understanding and experience or rating of pain as well as the demand for pain relief combined with the ever-increasing treatment options make SDM a key determinant of effective medical decision-making in APS. Additionally, efficiency and timely care around the clock must be provided in this special setting [22] asking for a systematic approach to guarantee personalized and patient-centred care at all times. While APS provide the organizational and technical basis for pain management, the complexity of such an “around-the-clock system” requires specific tools to ensure that patients receive the individualized, personalized therapy they expect. SDM should start at the pre-acute pain phase and go far beyond the subacute setting, accompanying the patient throughout the whole journey [22]. As a potential intervention, preoperative pain management through participatory decision-making can reduce postoperative opioid prescribing without compromising patient satisfaction [23]. Indeed, a preoperative SDM approach to postoperative opioid prescriptions led to a reduction in the number of unnecessarily prescribed opioids and an increase in patients who return the spare opioids they were given [23]. Thus, better education about the consequences of opioids and appropriate tools to support SDM in the APS setting can be beneficial to counteract an opioid crisis. It is through implementing tools like pre-emptive patient education, standardized and regular postoperative assessments, treatment adaptations according to patients’ preferences and prudent interdisciplinary planning that we can provide optimal individualized care for our patients [9•].

## SDM for APS Providers

For a start, identifying situations where SDM may be appropriate is relatively simple [24]: where alternative treatment methods exist and where conflict or ambiguity regarding available options arise, in problematic situations (multidimensional problems in individuals) or involvement of humanity-related aspects (self-identity, destruction of a person’s role due to a state of suffering) [25•], SDM is of great importance. All of those clinical challenges are encountered regularly in APS.

In the acute pain setting, the first encounter serves as the defining moment to establish a shared understanding of pain between patient and clinician and to develop a patient–physician relationship. Setting out patient expectations early in the treatment process is important, since patients have different preferences regarding the extent of involvement in their care [8, 26]. The MAPPIN’SDM [27] questionnaire is a useful tool to assess the needs and expectations of each patient regarding their involvement in the decision-making process (Table 1). Early expectation management allows for appropriate patient education. Training for techniques to modify the pain experience (e.g. music [28] or hypnosis [29, 30]) or further medical workup such as urine toxicology screening [9•] or laboratory analyses before peripheral nerve blocks [31] or wound infiltrations [28, 32] can be initiated in a timely manner. Meeting patient expectations at an early stage will later lead to increased patient satisfaction, an important outcome parameter [33]. Another useful clinical tool for this stage of the patient–physician interaction was developed by the Agency for Healthcare Research and Quality — the so-called seek, help, assess, reach, evaluate (SHARE) approach [6] (Table 2). The five steps of this approach may serve as a guidance during the early decision–making pathway in APS. Such a systematic approach to establish personalized “pain-relieving bundles” is the basis for effective patient-centred care at a later state of acute pain [34].

**Table 1:** Recommended tools for everyday clinical use of shared decision-making: SHARE (adapted from 6) MAGI (adapted from 45) and BRAN (adapted from 47)

**Tool**	**Official questions adapted for acute pain services**	**Sample discussion points for the example of making up a treatment plan for acute pain after shoulder surgery**
**SHARE**^6^	1. “Seek” patients’ participation	1. “There are many options that I would like to discuss with you”
	2. Offer “help” during the acquisition of knowledge regarding options for the treatment	2. “It is your decision. I am here to help you understand the options, including regional anesthesia, non-opioid and opioid drugs, local anesthetics applied intravenously and non-pharmacologic interventions”
	3. “Assess” values and preferences among patients	3. “What does pain mean to you, what degree of pain relief do you expect, which side effects are you willing to deal with, what route of administration works for you, how much control do you want to have over your treatment?” Some examples include fear of addiction, expectation to be able to self-medicate (e.g. via the use of patient-controlled anaesthesia), aversion regarding temporary palsy
	4. “Reach” a participatory decision	4. “After weighing out the pros and cons and based on your values, we chose a multimodal approach including regional anaesthesia and self-guided relaxation and meditative techniques for acute pain relieve. How are you feeling about that? We can talk about the decision again at any times”
	5. “Evaluate” the decision	5. Does the plan correspond to the medical problem (e.g. pain intensity, success rate and outcomes of the use of regional anaesthesia for this surgical setting) and emotional components (e.g. fear of addiction or fear of nerve injury or importance of not being handy capped by “motor blockade”)?
**MAGIC**^**45**^	1. What are the available options?	1. “I have intense shoulder pain, what are available treatment options?”
	2. Potential risks and benefits of the options available	2. “Are there any risks of applying opioids, non-opioids or regional anesthesia? Will it ease pain immediately?”
	3. How likely are the benefits and risks of each option to occur in my situation?	3. “Considering the fact that I have a history of gastric ulcers, how likely am I going to suffer from side effects of non-opioid drugs? Since immediate pain relief is important to me, how likely am I going to be satisfied with non-pharmacologic measures?”
**BRAN**^**47**^	**1. Benefits**	1. What benefits do I have from receiving regional anaesthesia?
	2. Risks	2. What are the risks involved with regional anaesthesia?
	3. Alternatives	3. Are there alternatives to this treatment and what are benefits/risks of those options? Using opioids, meditation/music/…, adjuvant drugs etc
	4. Consequences of no treatment	4. What happens if I do not seek treatment? Chronic pain, prolonged immobilization, etc

**Table 2:** Mappin’SDM (adopted from 44••)

MAPPIN’SDM^44^	1. Check that patients understood the medical problem that requires a decision
	2. Inquire about the clarity regarding the availability of multiple equipotent options, on which the medical provider cannot decide alone, without considering values, preferences and beliefs of each patient
	3. Check for accordance of the role distribution with each patients’ expectations during the consultation
	4. Inquire about patients’ awareness of the available treatment options
	5. Check whether benefits and risks are clear to the patients
	6. Evaluate the extent to which fears and expectations were addressed and included in the decision-making process
	7. Check whether the evidence supporting/opposing a decision is clear to patients
	8. Ask whether the information given by the provider was clear
	9. Inquire whether patients feel understood by the doctor
	10. Inquire whether patients were able to address remaining questions or uncertainties during the consultation
	11. Ask whether the final decision and the reasoning leading to this decision were clear to the patients at the end of the consultation

In the presence of acute pain, such as in the intra- and postoperative period or during an emergency room visit, without a previously established patient–physician relationship or anticipation of such a situation, participatory decision-making is difficult. There are various reasons for this: first, pain affects the decision-making capacity. Pain is perceived and cognitively integrated in different dimensions including affective motivational, sensory-discriminative and cognitive-evaluative, and both the cognitive and the emotional elements influence decision-making [35]. This results in a reduced decision-making capacity [36] and a tendency to make higher risk choices such as regarding treatment options or health-related behaviours [37].

Additionally, the current methods to acquire quantitative pain ratings in a state of acute pain are limited in their accuracy. There are various factors influencing the reporting of acute pain: for example, underrating happens by downplaying pain, anxiety-driven avoidance of “accepting” pain, attempts to reduce the required time in medical care or by actual avoidance of being given medication among many others. Also, adjustments to expectations of others in a sense of overrating pain, justification for seeking treatment and a lack of previous pain experiences or simply fear may influence pain ratings [38•]. Novel techniques such as quantitative sensory testing represent a valuable option to approach potential postoperative pain in personalized way [39, 40]. Still, decision-making in the context of acute pain can be very challenging.

This challenging situation requires more than a solid understanding of the underlying pathophysiology of the pain being experienced and of different methods for pain relief: excellent communication skills in the interaction with patients but also in the interdisciplinary setting and exceptional social skills to understand the individual’s needs and discuss the options available without bias. While these soft skills take time, experience and training to develop, the use of accurate language can easily be integrated: it is important, especially in the setting of imminent acute pain, to use numbers instead of words, to speak of absolute (not relative) risk, to frame symmetrically (e.g. say both: 20 out of 100 will develop complications after a certain intervention but also 80 out of 100 will have no problems) to use a pictorial format for illustration wherever possible [41]. In addition, the impact of positive expectations triggered by the wording used by the providers (so-called placebo wording) as opposed to worsening framings (nocebo) on pain is well-recognized [42]. After all, a physician-driven quick, unbiased assessment of individual needs, outweighing of available options and clear communication may enable SDM in the special setting of imminent acute pain.

Finally, as noted above, SDM starts at the first visit and goes far beyond the recovery room or discharge home after successful pain control. When aiming to optimize patient-reported outcome through the implementation of SDM, it is of paramount importance to inquire about the perceived experience in retrospect. This will allow APS specialist to learn and grow on an individual level but also to adapt current practices on a wider scale (organization-wide) according to the results of such questionnaires. The last question of the MAPPIN’SDM questionnaire mentioned above can serve as a valuable assessment tool regarding the needs and expectations of patients and in a second attempt to evaluate the individual’s satisfaction with the SDM process (Table 1). Apart from satisfaction, the Ottawa Decision Support Framework states immediate outcomes (quality of the decision) and longitudinal outcomes (feelings of regret) as key outcome parameters for SDM [43]. Advancements in SDM practice should be guided by the results of such patient (and physician) feedback tools.

## Empowering the Patient: Decision Aids in SDM

The willingness to participate in a decision is a state, not a trait [44••]. Thus, interventions to support patient-driven decision-making have been developed. Patients themselves may be encouraged to use accessible tools for knowledge acquisition even under time constraints. Such easy-to-use tools include the 3 “MAking Good decisions In Collaboration”(MAGIC) questions [45, 46] or the “Benefits, Risks, Alternatives and doing Nothing”(BRAN) tool [47] (Table 2). By adopting such tools for the APS setting, patient-driven concepts to acquire knowledge may enable SDM also in acute pain. Furthermore, decision coaching, clinical counselling or patient decision aids (PDAs) are gaining more and more attention [48, 49]. Of those, PDAs are the most widely used in other medical settings in an attempt to create optimally prepared patients for subsequent SDM. In the APS setting, the use of PDAs to inform about regional anaesthesia as a postoperative pain management option successfully increased active patient participation in the decision-making process when applied in pre-anaesthesia clinics [50]. While early PDAs came in the form of flyers, brochures or videos, nowadays, they are replaced by interactive programs in the form of online tools or web applications [51]. Technological advances allow for the delivery of individually tailored information that is readily available prior to the actual physician–patient interaction. Active participation in medical decision-making through increased patient knowledge or early clarification of individual values and thus reducing uncertainty and improving patient–physician communication may be achieved by the use of such tools [52]. While many PDAs exist for specific healthcare interventions or screening decisions, again, their development in the APS, such as for opioid prescriptions, lags behind [53]. One example of a successfully implemented PDA was the development of an online tool for the treatment of acute pain in juvenile idiopathic arthritis. It was given to the children and their parents and described different treatment options and the supporting evidence such as for Pilates, including text and pictures. Patients using the tool were able to make decisions regarding the management of pain more easily, reported increased knowledge of pain management options, better awareness of their own values and increased control of medical decision-making during interactions with practitioners. Also, non-beneficial interventions could be reduced [54]. Another innovative tool was developed for patients presenting to the emergency department with an acute injury, with a main focus on enhancing awareness of side effects of opioids. The online tool was handed out on admission before the patient–physician interaction and included a personal risk assessment, priority setting (e.g. which is more important to you “avoid addiction” vs “relieve pain”) and a tailored feedback report to be shared with the responsible team. Patients using the tool reported less conflict regarding the acute pain treatment decisions and felt more informed. However, the patients did not report a significantly higher SDM participation. The authors hypothesize that this might be due to a lack of teaching among the physicians such that the patients who were now aware of the options and of SDM in medical decision-making expected the subsequent consultation and SDM to be more participatory than the physicians were used to [53]. On the other hand, another tool that comes in the form of an app explaining the disease and possible therapeutic options to patients suffering from knee pain caused by osteoarthritis significantly improved patient knowledge and also increased patient satisfaction with involvement of care [55]. Thus, further studies assessing the role of PDAs in the APS setting and possible effects SDM participation and patient outcomes such as satisfaction are urgently needed.

After all, PDAs might be useful tools especially in the acute pain setting, where SDM is difficult due to impacts of pain on the patients’ cognitive capacity. Still, without proper education among practitioners they may not automatically lead to an improvement of SDM in APS.

## Remaining Challenges in Establishing an SDM Culture in APS

Recognized facilitators and barriers to successful SDM include anxiety, cultural background, trust and other psychodynamic factors [56]. In the APS setting, a lack of continuity of care or time and cost constraints may thus compromise participatory decision-making [9•] with enormous implications for the individual and the whole healthcare system as discussed above.

Another important issue regarding SDM is the fact that transferring knowledge from the clinician to the patient does not necessarily result in more participation for the patient in the decision-making process [57]. Power imbalances were reported as a key limiting factor in SDM [58••, 59, 60]. Perceptions as to whether a decision was made jointly or unilaterally differ between the doctor’s versus the patient’s perspectives [44, 61]. Unsuccessful SDM may have an impact on morbidity and mortality far beyond the individual decision [62, 63]. As an example, when it comes to the prescription of opioids, physicians may not be allowed to act according to the patient’s wishes. This may lead to treatment dis-adherence not only for the immediate complaint (pain) but also for other medical conditions [64, 65]. Knowledge regarding patients’ perception of SDM is still lacking.

Also, despite being driven by good intentions, there is evidence regarding biases concerning content and framing of information by physicians that may impact SDM [58••, 66]. As an example, according to the findings of a study conducted in patients undergoing thoracic surgery, anaesthesia providers tend to belittle risks of epidural analgesia during the preoperative visit. The authors attribute this attempt to direct the patients towards the application of such a neuraxial therapeutic modality to the common belief among anaesthesia providers that the knowledge and understanding needed to make a reasonable decision is lacking in patients. The patients, on the other hand, felt uninvolved in the decision-making process and disregarded in their needs. Still, in this study, the patients were not dissatisfied with the non-involvement in the decision-making process [67]. After all, this study shows that concerns regarding cognitive biases in SDM remain.

Apart from the actual patient–physician dyad, the organizational and system-level factors may be hinderers of SDM [68]. Current evidence supports the fact that appropriate framework conditions for SDM may be more important than the actual content of a participatory decision: open interaction, mutual respect, taking time, communicating in language that the patient can understand and forming a relationship that develops over a period of time were mentioned here [61]. A lack of private, calm spaces for conversations, of continuity of care or of the opportunity to organize multiple visits for a decision to be made are especially common barriers for SDM in APS, where patients are seen on various wards or in crowded recovery rooms by staff working on shift-based models [68]. Organizational changes are needed to create safe environments for SDM.

**Fig. 1:**
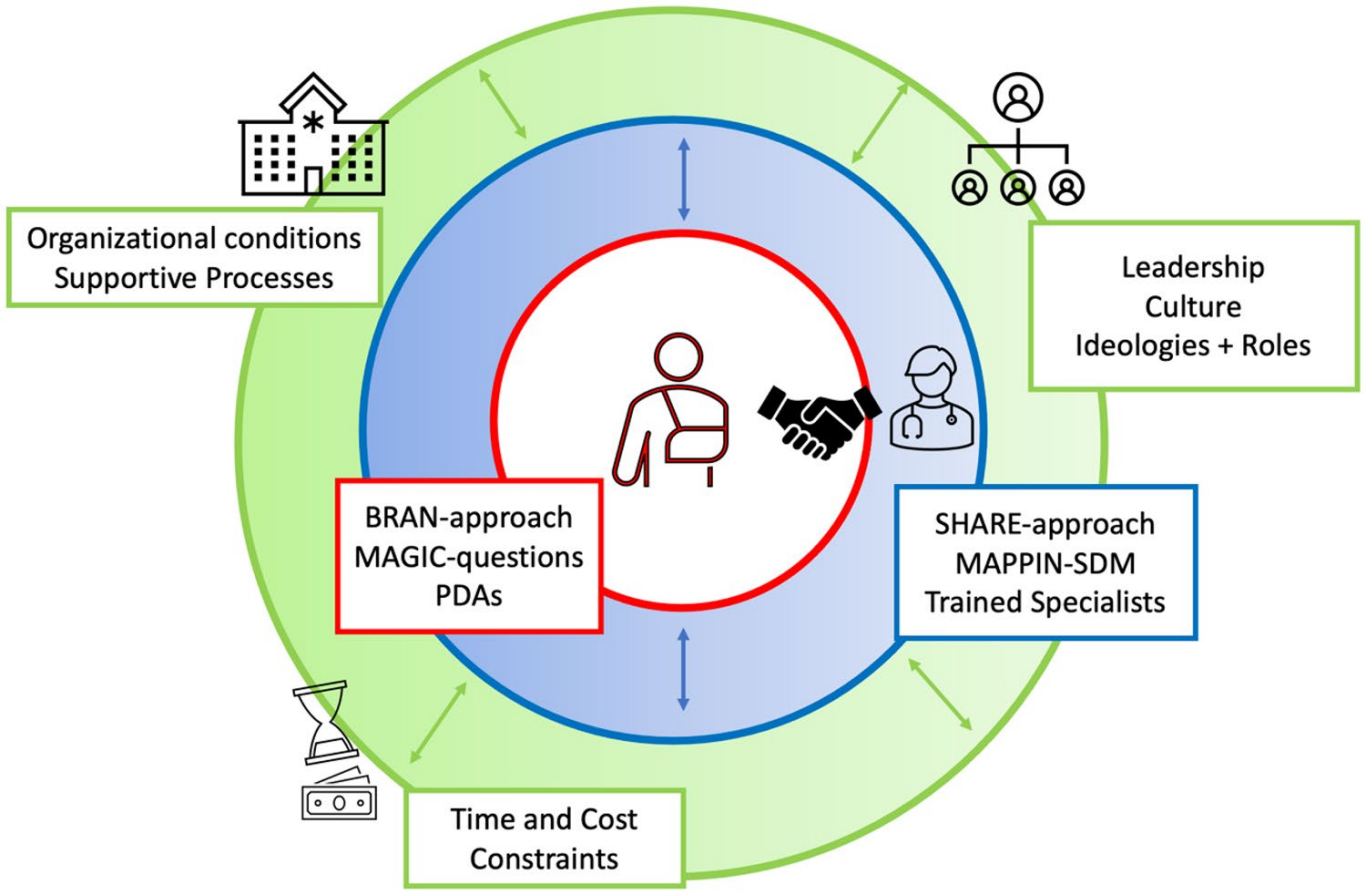
This figure illustrates the influencing factors for patient-centred care or more specifically shared decision-making (SDM) in the acute pain service (APS) setting. The patient–physician relationship is based on a mutual conversation to develop a common ground for the decision-making (represented by the hands shaking). The tools that may aid patients and physicians in reaching a consensus are depicted in the text red and blue boxes. Outside of this dyad are restrictions due to organizational conditions (e.g. lack of quiet rooms), discordant ideological view and roles (e.g. seeing physician as prestigious people), cultural and leadership conflicts with SDM (e.g. a reward culture based on the amount of patients seen or the amount of interventions performed), time and cost constraints that need to be changed to create suitable conditions for SDM in APS. BRAN, Benefits, Risks, Alternatives and doing Nothing; MAPPIN’SDM, multifocal approach to sharing in shared decision-making; PDA, patient decision aid; SHARE, seek, help, assess, reach, evaluate

Also, a lack of SDM skills in providers and conflicting expectations regarding the social and professional identity of the role of a physician versus the new role as a healthcare provider on par with the patient may be a burden for the establishment of SDM in APS [68]. Therefore, on top of organizational changes, encouraging education in patient-centred medicine for employees and especially APS trainees and specialists on a leadership level and thus promoting cultural changes are essential for the successful development of SDM in APS (Fig. 1).

The pressure of cost that many clinics are facing is another well-reported obstacle for the implementation of SDM [69]. Due to financial constraints, only large hospital can afford nursing staff or physicians specialized in pain medicine [9•]. APS generally do not generate money [9•]. Although the number of hospitals that operate an APS has increased over the past three decades [9•, 19, 70], the prevalence of postoperative pain is still underestimated and undertreated [70], with at least a fifth of the patients reporting severe pain within the first 24 h after surgery (some reports speak of more than 80% of cases where postoperative pain is not adequately managed [71]). Among surgical patients, 5–10% are at a high risk of developing chronic post-surgical pain –—a condition directly impacting quality of life [9•, 72-75]. According to current literature, a lack of well-organized APS in every hospital leads to excessive healthcare costs [76]. SDM increases knowledge, accuracy of risk perception and satisfaction [77] while minimizing inequalities, decisional conflict [77], provider cost [7], litigations and complaints [78, 79]. Developing APS that have a structured SDM concept will not directly add to an institution’s income but safe costs for the hospital and downstream national healthcare cost [9•]. This important value of APS is not reflected in the use of diagnosis-related groups and incentives for volume, thus asking for changes on the macrolevel. The definition of quality has to include patient-reported outcomes (complications, early oral feeding and ambulation, quality of life or satisfaction, among many others) [9•].

Finally, similarly to time and cost restraints, staff is a limited resource in current times [80-82]. The use of novel staffing models and involvement of acute pain physicians from remote might be a feasible solution to provide specialized care at any time and to any place [83-85]. Such an approach may extend the reach of APS and enable SDM for people with limited access to healthcare. After all, the benefits of SDM for staff must not be neglected, such as sharing the burden of responsibility for a decision with relatives, caregivers and the patients themselves [17] or relieving the pressure from unaligned values [86]. In addition, remote SDM may decrease workload and thus further prevent physician burnout [86-88]. How exactly SDM can be implemented in a remote setting remains a challenge that must be addressed.

## Conclusion

SDM as part of patient-centred care is an important yet not very well-established part of APS. Concepts such as the SHARE approach, the MAPPIN’SDM, the MAGIC question bundle or BRAN tool may guide SDM along the whole patient journey. PDAs have the potential to facilitate the implementation of SDM in everyday clinical practice. Nevertheless, such support tools require further investigation with regard to possible advantages such as time savings, patient satisfaction and economic benefits. Also, structured training for physicians to develop interventional skills and specialist knowledge regarding the complex management of acute pain, with an interdisciplinary thinking style and empathetic approach towards the patient, their families, caregivers and each member of the therapeutic team involved must be established. Finally, for a widespread establishment of SDM in APS, organizational changes are needed.

## Data Availability

Not applicable.

## Abbreviations

AHRQ: Agency for Healthcare, Research and Quality
APS: Acute pain services
BRAN: Benefits, Risks, Alternatives and doing Nothing
ED: Emergency department
MAGIC: Making Good decisions In Collaboration
MAPPIN’SDM: Multifocal approach to sharing in shared decision-making
NICE: National Institute for Health and Care Excellence
PDA: Patient decision aids
PRO: Patient-reported outcomes
SDM: Shared decision-making
SHARE: Seek, Help, Assess, Reach, Evaluate
